# Dengue virus induces PCSK9 expression to alter antiviral responses and disease outcomes

**DOI:** 10.1172/JCI137536

**Published:** 2020-08-31

**Authors:** Esther Shuyi Gan, Hwee Cheng Tan, Duyen Huynh Thi Le, Trieu Trung Huynh, Bridget Wills, Nabil G. Seidah, Eng Eong Ooi, Sophie Yacoub

**Affiliations:** 1 Duke-National University of Singapore Medical School, Singapore.; 2 Oxford University Clinical Research Unit (OUCRU), Ho Chi Minh City, Vietnam.; 3 Centre for Tropical Medicine and Global Health, University of Oxford, Oxford, United Kingdom.; 4 Laboratory of Biochemical Neuroendocrinology, Montreal Clinical Research Institute, Université de Montréal, Montréal, Québec, Canada.; 5 Saw Swee Hock School of Public Health, National University of Singapore, Singapore.; 6 SingHealth Duke–National University of Singapore Global Health Institute, Singapore.; 7 Antimicrobial Resistance Interdisciplinary Research Group, Singapore MIT Alliance in Research and Technology, Singapore.

**Keywords:** Infectious disease, hypoxia

## Abstract

Dengue virus (DENV) infection requires cholesterol as a proviral factor, although statin treatment did not show antiviral efficacy in patients with dengue. Here, we show that DENV infection manipulated cholesterol metabolism in cells residing in low-oxygen microenvironments (hypoxia) such as in the liver, spleen, and lymph nodes. DENV infection induced expression of proprotein convertase subtilisin/kexin type 9 (*PCSK9*), which reduces low-density lipoprotein receptor (*LDLR*) recycling and hence cholesterol uptake. We found that, whereas *LDLR* uptake would have distributed cholesterol throughout the various cell compartments, de novo cholesterol synthesis enriched this lipid in the endoplasmic reticulum (ER). With cholesterol enrichment in the ER, ER-resident STING and type I IFN (*IFN*) activation was repressed during DENV infection. Our in vitro findings were further supported by the detection of elevated plasma PCSK9 levels in patients with dengue with high viremia and increased severity of plasma leakage. Our findings therefore suggest that PCSK9 plays a hitherto unrecognized role in dengue pathogenesis and that PCSK9 inhibitors could be a suitable host-directed treatment for patients with dengue.

## Graphical abstract

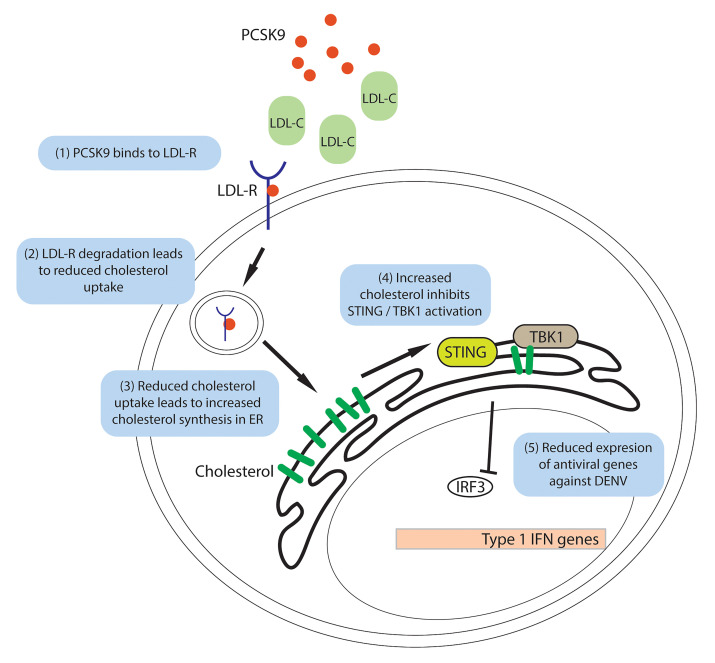

## Introduction

Dengue is a major global health problem. Transmitted by the urban-adapted *Aedes* mosquitoes, an estimated 390 million individuals are infected with 1 of the 4 types of dengue virus (DENV) annually (1). Infected individuals present with a range of clinical signs and symptoms, from asymptomatic infection, to self-limiting but debilitating acute febrile illness, to severe dengue characterized by hypovolemic shock from vascular leakage, organ dysfunction, and internal bleeding (2). If not properly managed, severe dengue disease can result in a mortality rate of up to 20% (3). Dengue prevention thus far has relied on vector population suppression, which, when conducted comprehensively, is costly and lacks long-term sustainability (4). A dengue vaccine, DENGVAXIA, has been licensed, although it is only indicated for individuals who have had a prior DENV infection. DENGVAXIA, paradoxically, enhances DENV infection in those who are immunologically naive at vaccination and can therefore only be given to individuals with evidence of prior DENV infection (5). No licensed antiviral drug is available to treat dengue. These limitations collectively hamper our ability to reduce the global burden of dengue.

Functional genomics and studies on dengue pathogenesis have identified host factors upon which DENV depends for successful infection (6–10). These findings have collectively raised the possibility of repurposing licensed inhibitors of such host factors as antiviral therapies. Such a strategy would reduce the long lead time and costs associated with new drug discovery. One such host factor is cholesterol. DENV interacts with host cellular membranes for multiple and critical steps of its life cycle — viral entry, fusion, and replication (11). The composition of cellular membranes, especially cholesterol content, has thus been found to affect DENV infection. Previous in vitro studies have shown that DENV stimulates host cells to increase the synthesis of intracellular cholesterol by upregulating the enzymatic activity of 3-hydroxy-3-methylglutaryl-coenzyme A (*HMG-CoA*) reductase (12). As de novo cholesterol synthesis occurs in the endoplasmic reticulum (ER) (13), cholesterol aids in restructuring the ER to enable the formation of replication complexes where DENV reproduces (11). Reducing de novo cholesterol synthesis by using statins to inhibit the rate-limiting HMG-CoA reductase activity therefore reduced DENV infection, both in vitro (14, 15) and in animal models (16). Despite these promising preclinical findings, however, a clinical trial of the HMG-CoA reductase inhibitor lovastatin failed to show useful benefit in patients with dengue (17). This negative clinical trial finding thus brings into question the clinical validity of the laboratory findings.

The discrepant outcomes between laboratory-based studies and the clinical trial may be due to oxygen and its impact on cholesterol metabolism. DENV replicates in the liver, spleen, and lymph nodes (18–21). These organs are known to have lower oxygen levels (~3%–5% oxygen) as a consequence of the circulatory anatomy (22). Reduced oxygenation would alter cholesterol homeostasis (23), although how such altered cholesterol homeostasis affects DENV infection and pathogenesis is unknown. Such hypoxia-driven changes in cholesterol homeostasis may underpin the lack of efficacy of statins as an anti-dengue drug. Therefore, further investigations into these hypoxia-driven changes during DENV infection can provide alternative therapeutic targets such as proprotein convertase subtilisin/kexin type 9 (PCSK9), a key regulator of cholesterol metabolism (24).

Here, we show that DENV infection induces the expression of *PCSK9*, a negative regulator of the low-density lipoprotein receptor (*LDLR*). Increased PCSK9 levels in hypoxic cells reduced *LDLR* and hence LDL cholesterol (LDL-C) uptake, which further drove de novo cholesterol synthesis. Whereas *LDLR* cholesterol uptake would have distributed cholesterol throughout the cell, de novo cholesterol synthesis enriched ER cholesterol levels that suppressed the phosphorylation of stimulator of IFN gene (*STING*) and tank-binding kinase (*TBK*) despite DENV infection. Reduced STING and TBK activation, in turn, lowered the expression of type I IFN (*IFN*) and the downstream antiviral IFN-stimulated genes (ISGs). These in vitro findings were supported by clinical data that showed a direct correlation between plasma levels of PCSK9 with higher viremia levels and disease severity in patients with dengue. These findings also indicate that PCSK9 is a host factor for DENV in target cells resident in hypoxic microenvironments and that inhibiting PCSK9 (25) rather than just *HMGCoA* reductase could be a useful approach to fill the therapeutic void for dengue treatment.

## Results

### DENV alters LDLR and PCSK9 expression under hypoxic conditions.

**Figure 1:**
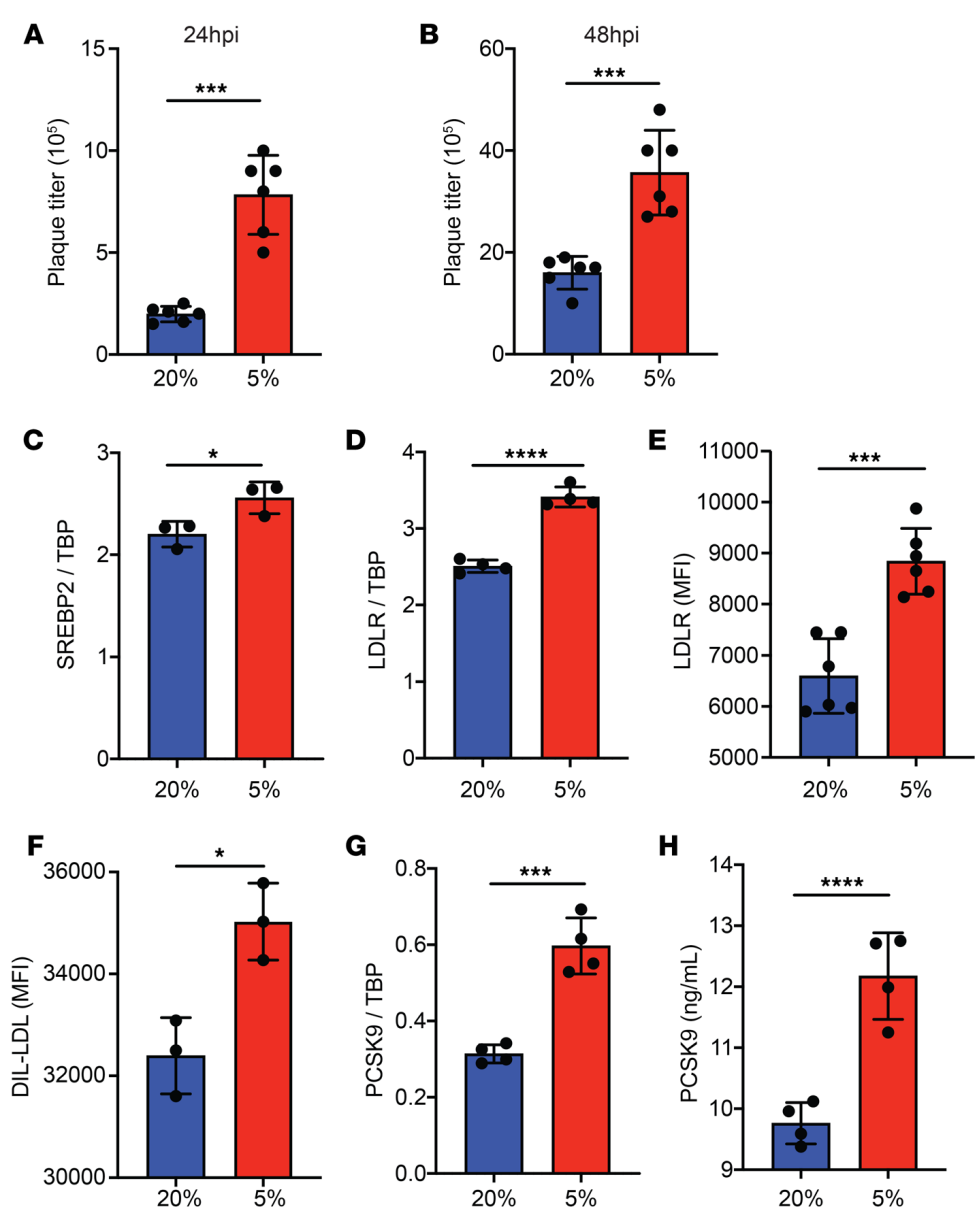
Hypoxia and DENV infection both alter LDLR and PCSK9 expression. (**A** and **B**) Plaque titers from normoxic (blue) and hypoxic (red) Huh7 cells 24 (**A**) and 48 (**B**) hours after DENV2 infection. Data are expressed as PFU per milliliter of culture supernatant. (**C**) *SREBP2* mRNA levels in normoxic (blue) and hypoxic (red) Huh7 cells after 24 hours’ incubation. (**D**) *LDLR* mRNA levels in normoxic (blue) and hypoxic (red) Huh7 cells 24 hours after oxygen adaptation. (**E**) MFI of *LDLR* in normoxic (blue) and hypoxic (red) Huh7 cells 24 hours after oxygen adaptation as assessed by flow cytometry. (**F**) MFI of DIL-LDL in normoxic (blue) and hypoxic (red) Huh7 cells 24 hours after oxygen adaptation as assessed by flow cytometry. (**G**) *PCSK9* mRNA expression in normoxic (blue) and hypoxic (red) Huh7 cells 24 hours after oxygen adaptation. (**H**) Levels of secreted PCSK9 in normoxic (blue) and hypoxic (red) Huh7 cells 24 hours after oxygen adaptation as measured by ELISA. Experiments were replicated 3 times, each with a minimum of 3 biological replicates. Representative data from 1 of these 3 independent experiments are shown. Data in **A**–**H** represent the mean ± SD. *P < 0.05, ***P < 0.001, and ****P < 0.0001, by unpaired *t* test.

DENV has been found to infect and replicate in myeloid-derived cells in lymph nodes and the spleen as well as in hepatocytes (26). All these organs have hypoxic microenvironments. We previously observed that monocytes cultured at 3% O_2_ resulted in increased DENV infection (27). As liver-derived Huh7 cells are more susceptible to in vitro DENV infection than are monocytic cell lines, we first sought to determine the response of Huh7 cells to incubation at 5% O_2_. In uninfected cells, incubation at 5% O_2_ (hereafter referred to as hypoxia) for 24 hours produced the known transcriptional response to hypoxia and corresponding changes in cholesterol metabolism. We detected increased expression of hypoxia-induced genes such as adrenomedullin (*ADM*), vascular endothelial growth factor (*VEGF*), and glucose transporter 1 (*GLUT1*) (ref. 28 and Supplemental Figure 1, A–C; supplemental material available online with this article; https://doi.org/10.1172/JCI137536DS1) in Huh7 cells incubated in hypoxic versus normoxic conditions. With DENV infection, hypoxic Huh7 cells produced higher DENV plaque titers than did normoxic cells (Figure 1, A and B), suggesting that hypoxia induced proviral changes in Huh7 cells as well.

Hypoxia has previously been shown to alter cholesterol metabolism pathways (29). In uninfected Huh7 cells, expression of *SREBP2*, the master regulator of sterol synthesis, was upregulated, as expected (29, 30), when incubated under hypoxic versus normoxic conditions (Figure 1C). Likewise, the SREBP2-regulated *LDLR* (Figure 1, D and E) was similarly induced in hypoxic Huh7 cells and resulted in increased LDL uptake (Figure 1F). *PCSK9*, the negative regulator of *LDLR*, was also induced (Figure 1, G and H) and was likely to ensure tight regulation of intracellular cholesterol levels (31, 32).

**Figure 2:**
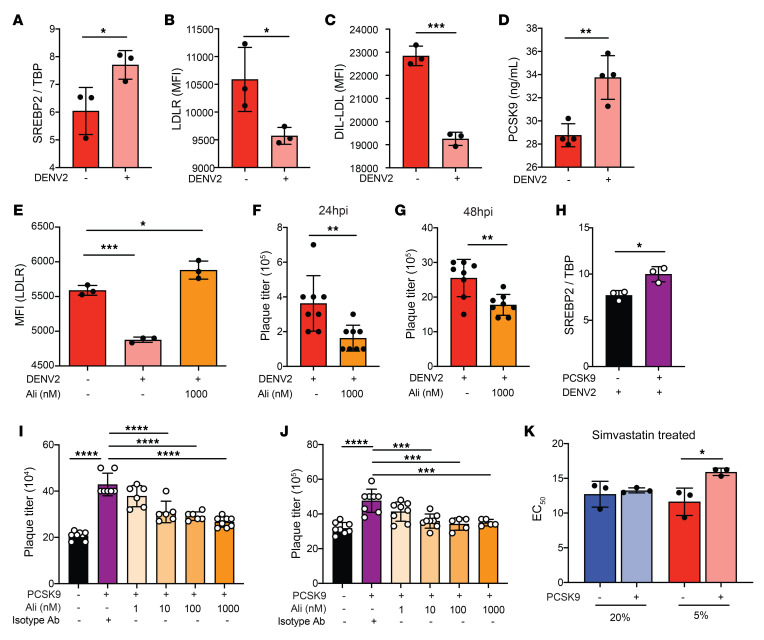
PCSK9 augments DENV infection and dampens the antiviral effect of statins. (**A**) *SREBP2* mRNA levels in uninfected and infected hypoxic Huh7 cells 24 hpi. (**B**) MFI of *LDLR* in uninfected and infected hypoxic Huh7 cells 24 hpi as assessed by flow cytometry. (**C**) MFI of DIL-LDL in uninfected and infected hypoxic Huh7 cells 24 hpi as assessed by flow cytometry. (**D**) Levels of secreted PCSK9 in uninfected and infected hypoxic Huh7 cells 24 hpi. (**E**) MFI of LDL in uninfected, infected, and alirocumab-treated (Ali) hypoxic Huh7 cells 24 hpi as assessed by flow cytometry. (**F** and **G**) Plaque titers in hypoxic Huh7 cells treated with or without alirocumab 24 (**F**) and 48 (**G**) hpi. (**H**) mRNA expression of *SREBP2* in hypoxic Huh7 cells 6 hpi. Cells were cultured without (black) or with supplementation of 400 ng/mL PCSK9 (purple) 24 hours before DENV2 infection. (**I** and **J**) Plaque titers in hypoxic Huh7 cells 24 (**G**) and 48 (**H**) hpi with DENV2 infection. Cells were cultured without (black), with supplementation of 400 ng/mL PCSK9 (purple), or with PCSK9 with increasing doses of alirocumab (orange) for 24 hours before DENV2 infection. (**K**) EC_50_ values of hypoxic (red) and normoxic (blue) Huh7 cells 48 hpi with DENV2 infection with varying doses of simvastatin. Cells were cultured without (dark blue, dark red) or with supplementation of 400 ng/mL PCSK9 (light blue, light red) 24 hours before DENV2 infection. Experiments were replicated 3 times, with a minimum of 3 biological replicates. Representative data from 1 of these 3 independent experiments are shown. Data in **A**–**K** represent the mean ± SD. *P < 0.05, **P < 0.01, ***P < 0.001, and ****P < 0.0001, by unpaired *t* test (**A**–**D** and **F**–**H**) and 1-way ANOVA corrected for multiple comparisons (**E** and **I**–**K**).

Upon DENV infection, *SREBP2* expression was further augmented in hypoxic Huh7 cells (Figure 2A). However, DENV infection under hypoxic conditions resulted in significantly reduced plasma membrane *LDLR* levels and LDL 24 hours after infection (Figure 2, B and C). In contrast, DENV-infected cells showed a further increase in PCSK9 secretion (Figure 2D). As *LDLR* expression can be altered at posttranslational stages via its negative regulator *PCSK9* (31–33), we examined whether reduced *LDLR* was due to the function of increased PCSK9 secretion. We treated cells with alirocumab, a therapeutic mAb against PCSK9 (34, 35). Compared with mock-treated cells, alirocumab treatment restored plasma membrane levels of *LDLR* in DENV-infected cells (Figure 2E) and resulted in lower DENV plaque titers 24 and 48 hours post infection (hpi) (Figure 2, F and G). These findings collectively suggest that, while cholesterol uptake through increased *LDLR* occurred as expected in Huh7 cells incubated under hypoxic conditions, DENV infection downregulated LDLR protein levels via increased expression of *PCSK9*.

### PCSK9 augments DENV infection in Huh7 cells.

To further define the role that PCSK9 plays in DENV infection, we supplemented Huh7 cell cultures with recombinant PCSK9 before infection. The PCSK9 concentration that maximally reduced *LDLR* levels was determined by incubating cells with a range of PCSK9 concentrations for 24 hours (Supplemental Figure 2A). We found that 400 ng/mL PCSK9 resulted in a maximal decrease in plasma membrane *LDLR* expression under hypoxic conditions. As expected, PCSK9 supplementation increased *SREBP2* mRNA expression 6 hpi in DENV-infected Huh7 cells (Figure 2H), resulting in increased DENV plaque titers 24 hpi (Figure 2I) and 48 hpi (Figure 2J). This effect could be inhibited with the addition of alirocumab (Figure 2, I and J) in a dose-dependent manner that reduced *LDLR* expression (Supplemental Figure 2B). However, the effect of PCSK9 on DENV infection was specific to hypoxic cells, as a similar supplementation experiment in normoxic Huh7 cells did not result in similar outcomes (Supplemental Figure 2C). The PCSK9-induced increase in DENV infection was not limited to DENV2 infection, as similar findings were also observed with DENV1, DENV3, and DENV4 (Supplemental Figure 3, A–C). Collectively, these results indicate that PCSK9 reduces LDLR-mediated cholesterol uptake in DENV-infected hypoxic cells.

### Increased PCSK9 activity may account for the lack of antiviral efficacy of statins.

Our finding of a PCSK9-mediated reduction in *LDLR* levels, in a background of hypoxia-induced *SREBP2* expression, also suggested that cholesterol synthesis in DENV-infected cells could be further increased. This PCSK9 activity may have rendered standard doses of statins ineffective. To test this possibility, we first treated Huh7 cells with simvastatin or mevastatin, with and without PCSK9 supplementation. Under normoxia, nontoxic doses (Supplemental Figure 4, A and B) produced no difference in EC_50_ between PCSK9-supplemented and nonsupplemented cells (Figure 2K and Supplemental Figure 4, C and E). Under hypoxia, however, we found that PCSK9 supplementation reduced the potency of both simvastatin and mevastatin in reducing DENV titers (Figure 2K and Supplemental Figure 4, D and F).

### PCSK9 augments DENV infection in primary myeloid cells.

**Figure 3:**
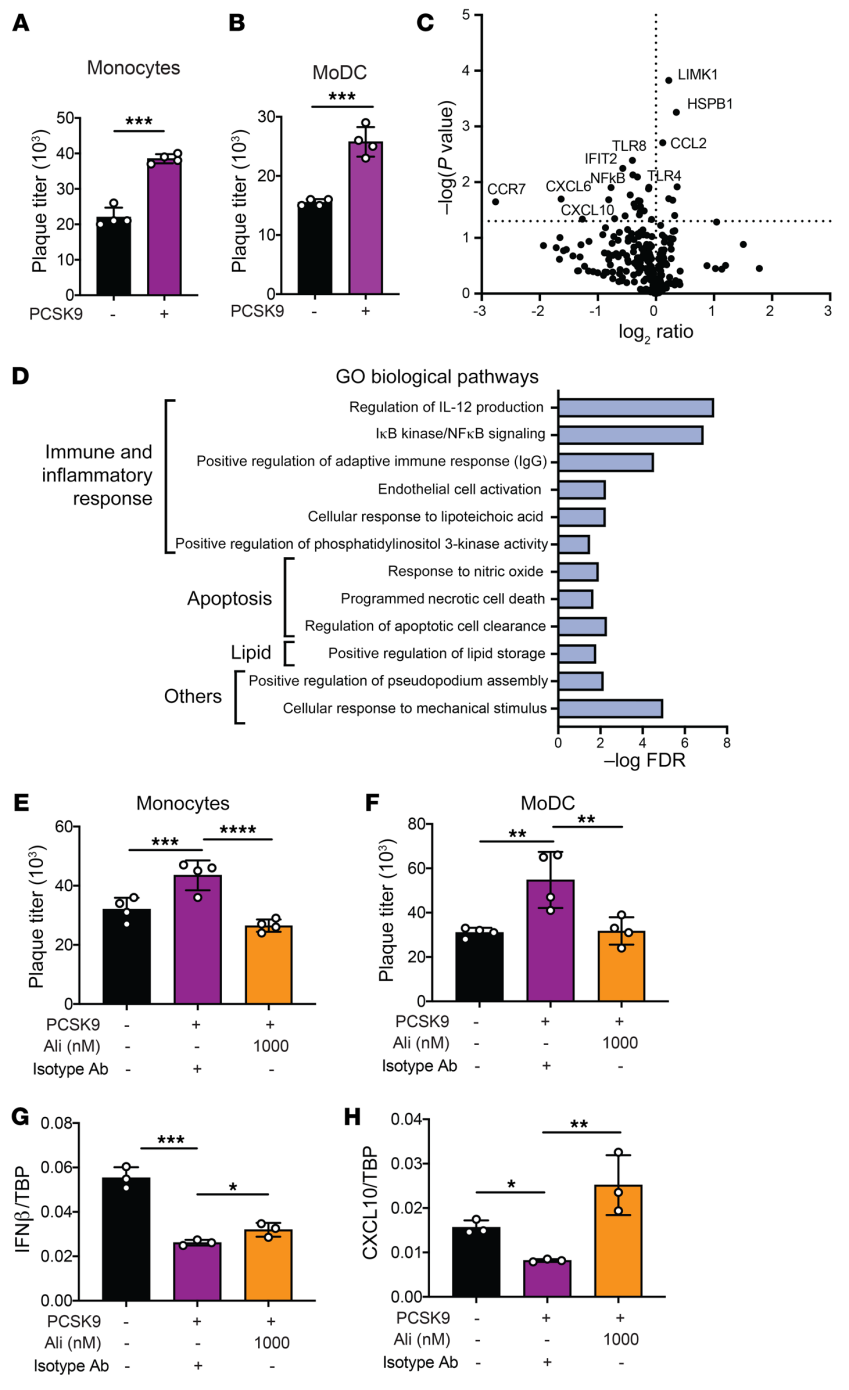
PCSK9 augments DENV infection in primary myeloid cells. (**A**) Plaque titers for hypoxic primary monocytes 48 hpi with DENV infection. Cells were cultured without (black) or with supplementation of 400 ng/mL PCSK9 (purple) 24 hours before DENV2 infection. (**B**) Plaque titers for hypoxic primary MoDCs 48 hpi with DENV infection. Cells were cultured without (black) or with supplementation of 400 ng/mL PCSK9 (purple) 24 hours before DENV2 infection. (**C**) Volcano plot displaying 245 genes detected by NanoString in primary monocytes 24 hpi with DENV2. (**D**) Pathway analysis of genes that were most abundantly downregulated in PCSK9-supplemented primary monocytes as compared with nonsupplemented cells 24 hpi with DENV2 infection. Downregulated genes were analyzed against the GO biological pathway analysis and further summarized via REVIGO web server. (**E**) Plaque titers of hypoxic primary monocytes 48 hours after DENV2 infection. Cells were cultured without PCSK9 (black), with supplementation of 400 ng/mL PCSK9 (purple), or with PCSK9 and alirocumab (orange). (**F**) Plaque titers of hypoxic primary MoDCs 48 hours after DENV infection. Cells were cultured without PCSK9 (black), with supplementation of 400 ng/mL PCSK9 (purple), or with PCSK9 and alirocumab (orange). (**G** and **H**) mRNA expression of *IFNB* (**D**) and *CXCL10* (**E**) in hypoxic MoDCs without (black) or with PCSK9 supplementation (purple) 6 hours after DENV2 infection. Experiments in **A**, **B**, **E**, and **H** were replicated 3 times, each with a minimum of 3 biological replicates. Representative data from 1 of these 3 independent experiments are shown. *P < 0.05, **P < 0.01, ***P < 0.001, and ****P < 0.0001, by unpaired *t* test (**A** and **B**) and 1-way ANOVA corrected for multiple comparisons (**E**–**H**). Data in **A**, **B**, and **E**–**H** represent the mean ± SD.

As myeloid cells are primary targets of DENV, we next determined whether the above findings could be replicated in primary human monocytes and monocyte-derived dendritic cells (MoDCs). At 3% O_2_, which more closely simulates the O_2_ microenvironment of lymph nodes (36), Supplementation of primary monocytes and MoDCs produced higher DENV titers following infection (Figure 3, A and B). Gene expression analyses of DENV-infected, PCSK9-supplemented primary monocytes using the 250-gene human inflammation panel on the NanoString nCounter platform revealed 7 marginally upregulated and 23 significantly downregulated genes (Figure 3C). Gene Ontology (GO) biological pathway analysis identified significant downregulation of the *NFKB* and *IFN* pathways (Figure 3D). These findings suggest that supplementation of PCSK9 dampens the antiviral response against DENV, which could account for the observed increase in DENV replication. These changes in DENV replication (Figure 3, E and F) and antiviral responses such as IFNβ and C-X-C motif chemokine 10 (*CXCL10*) (Figure 3, G and H) were abrogated with the addition of alirocumab, indicating that these effects were specific to increases in PCSK9 concentrations. Collectively, our findings suggest that increased *PCSK9* expression augments DENV infection in human myeloid-derived cells by reducing antiviral responses under low-oxygen conditions representative of lymph node microenvironments.

### PCSK9 suppresses STING activation and the downstream antiviral response.

The above findings suggest that DENV derives replicative advantage from PCSK9-mediated cellular responses. A clue for what these responses could be came from a recent study that showed increased *STING* and *TBK* activation upon reduced cholesterol levels in the ER (37). Since ER cholesterol enrichment is dependent on de novo cholesterol synthesis (38), whereas LDLR-mediated uptake distributes cholesterol throughout the cell (39), we hypothesized that DENV depends on de novo cholesterol synthesis to inhibit *STING* and *TBK* activation.

**Figure 4:**
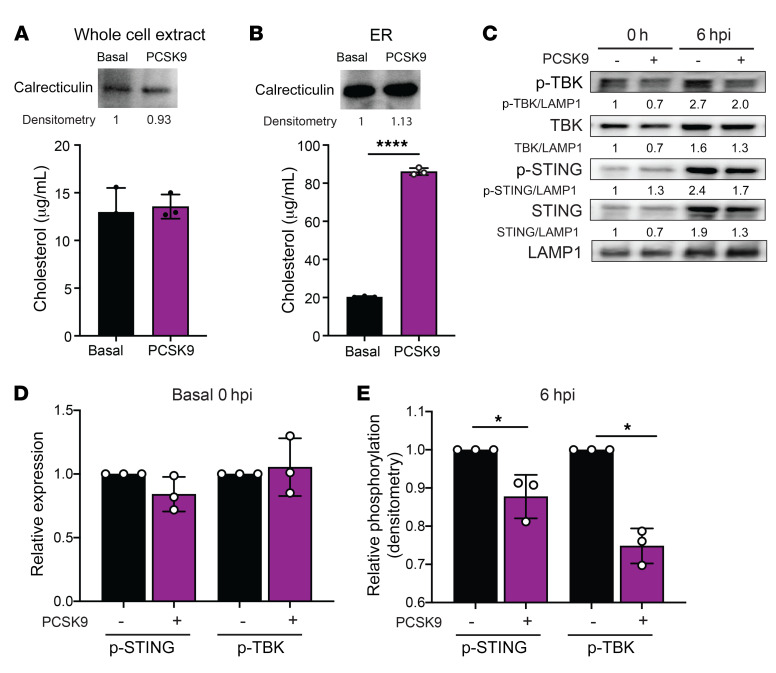
PCSK9 suppresses STING-dependent induction of type-I IFN during DENV infection. (**A**) ER cholesterol quantification of whole-cell extract of Huh7 cells with (purple) and without (black) PCSK9 supplementation. (**B**) ER cholesterol quantification of ER organelles from Huh7 cells with (purple) and without (black) PCSK9 supplementation. (**C**) Representative Western blot of levels of p-TBK, TBK, p-STING, STING and LAMP1 with or without PCSK9 supplementation in Huh7 cells, 0 and 6 hours after DENV2 infection. LAMP1 served as a loading control. Numbers under each Western blot indicate the levels of each protein normalized to LAMP1 and relative to Huh7 cells without PCSK9 supplementation at 0 hpi. (See the complete unedited blots in the supplemental material.) (**D** and **E**) DENV infection samples in Figure 3C were analyzed 3 times as shown by densitometry. Relative phosphorylation represents the levels of each protein normalized to LAMP1 and relative to Huh7 cells without PCSK9 supplementation, 0 (**D**) and 6 (**E**) hours after DENV2 infection. Experiments were replicated 3 times, each with a minimum of 3 biological replicates. Representative data from 1 of these 3 independent experiments are shown. Data in **A**, **B**, **D**, and **E** represent the mean ± SD. *P < 0.05 and ****P < 0.0001, by unpaired *t* test.

To determine whether de novo cholesterol synthesis resulted in enriched ER cholesterol levels, we fragmented hypoxic, PCSK9-supplemented and nonsupplemented Huh7 cells and isolated the ER. Although total cellular cholesterol levels were similar in cells grown under both conditions (Figure 4A), ER cholesterol levels were enriched in hypoxic, PCSK9-supplemented cells (Figure 4B). We next examined the impact of cholesterol enrichment in the ER on *STING* and *TBK* activation. At baseline, PCSK9 supplementation did not result in significant differences in STING, phosphorylated STING (p-STING), TBK, or p-TBK levels (Figure 4, C and D). With DENV infection, however, levels of both p-STING and p-TBK were significantly lower 6 hpi in PCSK9-supplemented Huh7 cells compared with levels in nonsupplemented controls (Figure 4, C and E). STING and TBK activation is known to phosphorylate and activate IFN regulatory factor 3 (IRF3), leading to induction of type-I IFN activation. Indeed, PCSK9 supplementation in Huh7 cells reduced the expression of *IFNB* and ISGs, such as *CXCL10* and *MX1*, which were all reversed with the addition of alirocumab (Supplemental Figure 5, A–C). Collectively, these data suggest that *PCSK9* expression reduced LDLR-mediated cholesterol uptake to induce de novo cholesterol synthesis, which enriched ER cholesterol levels that in turn impaired STING-mediated type-I IFN induction.

### Plasma PCSK9 concentrations increase in patients with severe dengue.

**Figure 5:**
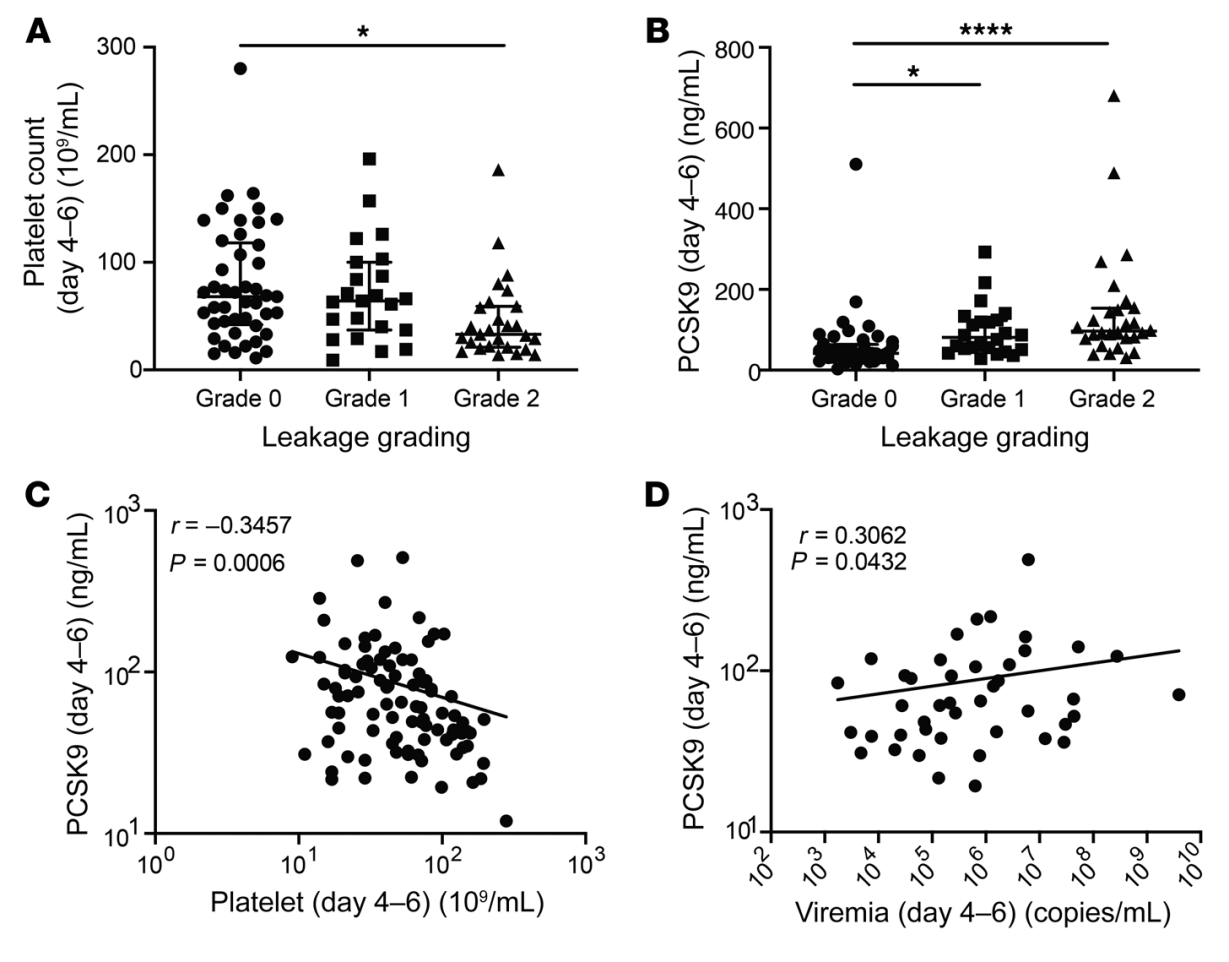
Increased plasma PCSK9 concentrations is associated with higher viremia, a greater extent of thrombocytopenia, and plasma leakage in patients with dengue. (**A**) Platelet counts of patients 4–6 days after onset of illness, categorized by disease severity. (**B**) PCSK9 concentrations in patients 4–6 days after onset of illness, categorized by disease severity. (**C**) Spearman’s correlation of PCSK9 and platelet counts of patients 4–6 days after onset of illness. Normal platelet counts in patients ranged from 150 × 10^9^/L to 400 × 10^9^/L. (**D**) Spearman’s correlation of PCSK9 and plasma viremia levels 4–6 days after onset of illness. Data in **A** and **B** represent the mean ± SD. *P < 0.05 and ****P < 0.000, by 1-way ANOVA corrected for multiple comparisons (**A** and **B**) and Spearman’s correlation test (**C** and **D**).

To verify that our in vitro findings were clinically pertinent, we examined the association between plasma PCSK9 levels, DENV viremia, and disease severity in a nested case-control study using a previously described prospectively enrolled cohort of patients with dengue (40). A total of 314 patients with suspected dengue were enrolled at 2 Vietnamese hospitals, either as outpatients with less than 72 hours of fever, or after hospitalization. Of these, 263 patients were laboratory-confirmed dengue cases (ref. 40 and Table 1). A subset of 111 individuals with good clinical and basic laboratory data (serial measurements of platelet, WBC, neutrophil and lymphocyte counts in whole blood, serum aspartate aminotransferase [AST] and alanine transaminase [ALT] levels, as well as plasma viremia, as measured by quantitative reverse transcription PCR [qRT-PCR] at enrollment) and availability of residual plasma for PCSK9 measurements were included in this analysis. PCSK9 levels in each patient were measured at 3 time points (1–3 days after illness onset, 4–6 days after illness onset and at convalescence, and 10–14 days after illness onset) (Table 2). Patients were classified into 3 predefined categories of increasing disease severity in terms of plasma leakage: grade 0, no evidence of leakage; grade 1, an increase of 15%–20% in hematocrit (HCT) and/or fluid accumulation and; grade 2, development of severe leakage including an HCT increase of more than 20%, dengue shock syndrome (DSS), or compromised respiratory function. Relevant clinical details on the 111 patients analyzed in this study are provided in Table 1. Consistent with previous findings, platelet counts were significantly lower in patients with grade 2 compared with grade 0 plasma leakage 4–6 days after illness onset, which is around the period of defervescence when signs of severe dengue commonly manifest (Figure 5A).

Plasma PCSK9 levels on illness days 1–3 were similar among all patients (Table 2). However, the mean levels of PCSK9 in patients with grade 0, 1, or 2 dengue on illness days 4–6 after illness onset were 46.11 ng/mL, 95.76 ng/mL, and 145.8 ng/mL, respectively, indicating elevated PCSK9 levels in patients with more severe disease (Figure 5B). Plasma PCSK9 concentrations were negatively correlated with platelet counts (Figure 5C) and positively correlated with viremia levels (Figure 5D) at this time point. Collectively, these findings indicate that elevated PCSK9 expression is associated with higher viremia and an increased risk of more severe plasma leakage in patients with dengue.

## Discussion

Cholesterol plays proviral roles in DENV infection. Previous studies have shown that cholesterol is required for efficient DENV replication and egress. The lack of efficacy in the use of statins as an antiviral treatment against dengue was thus unexpected and underscores the need for a more detailed and clinically pertinent understanding of the interaction between DENV and cellular cholesterol.

This study highlights a hitherto unrecognized role of PCSK9 in shaping cholesterol homeostasis in hypoxic cells to favor DENV infection. DENV infection in low-oxygen microenvironments upregulates the expression and secretion of PCSK9, a negative regulator of *LDLR*. Without PCSK9, LDLR–LDL-C complexes are internalized via endocytosis. The acidic pH of endosomes releases LDL-C and induces a conformational change in the extracellular domain of *LDLR*, aiding in its recycling to the plasma membrane (41). PCSK9, however, binds and inhibits this conformational change in *LDLR*, preventing its recycling. In such an instance, *LDLR* is routed to lysosomes for degradation, thus lowering its surface expression levels (42) and plasma cholesterol uptake.

With reduced *LDLR* expression through the increased activity of *PCSK9*, *SREBP2* would be further induced to drive expression of cholesterol biosynthesis enzymes (38, 43). Cholesterol synthesis occurs and accumulates in the ER before its distribution to subcellular pools, including the plasma membrane. Our data suggest that increased de novo cholesterol synthesis resulted in the cholesterol enrichment in the ER that inhibited ER-resident *STING* and *TBK* activation and hence downstream type-I IFN responses. Furthermore, we demonstrate that upregulation of cholesterol synthesis by PCSK9-dependent reductions in LDLR-mediated cholesterol uptake could have, at least in part, reduced the anti-DENV efficacy of statins (15). Thus, we suggest that the subcellular localization of cholesterol, rather than total cellular cholesterol levels, is the proviral determinant of DENV infection.

Although we have used alirocumab to demonstrate a functional role for PCSK9 in DENV infection, our findings suggest an anti-dengue potential for anti-PCSK9 inhibitors. PCSK9 inhibitors have been developed primarily as an alternative therapeutic approach to treat hypercholesterolemia. Two anti-PCSK9 mAbs, alirocumab (Praluent, Sanofi/Regeneron Pharmaceuticals) (34, 44) and evolucumab (Repatha, Amgen) (45, 46), have been licensed as treatments to lower plasma cholesterol. Both alirocumab and evolucumab are fully humanized mAbs that bind to free plasma PCSK9 and promote the degradation of its target. Reduced PCSK9 levels allow *LDLR* to be recycled back to the plasma membranes after endocytosis, thereby increasing the expression of PCSK9. Clinically, both Abs have shown remarkable efficacy in reducing both plasma PCSK9 and cholesterol levels (45, 47).

Given the possibility that PCSK9 inhibitors could be repurposed as anti-dengue therapy, we thus sought to establish an association between plasma PCSK9 levels and disease severity in patients with dengue. Viremia and NS1 antigenemia levels have been found to be directly correlated with dengue severity (48–52). Greater viral and NS1 antigen burdens during infection, both directly and indirectly through proinflammatory responses, are thought to compromise the integrity of the vascular endothelium, leading to plasma leakage (53–55). That the plasma PCSK9 levels were directly correlated with viremia levels and the extent of plasma leakage as well as negatively correlated with platelet counts collectively suggest that our in vitro findings are clinically pertinent and that PCSK9 is involved in clinical pathogenesis. Repurposing PCSK9 inhibitors, either on their own or in conjunction with statins, could thus be an expedient approach to fill the therapeutic void for dengue treatment.

Although our study highlights an important role that *PCSK9* plays in DENV infection, a limitation is the concentrations of recombinant PCSK9 (rPCSK9) supplemented in our in vitro experiments. Concentrations of 400 ng/mL PCSK9 supplemented in vitro were higher than the levels of plasma PCSK9 observed in our clinical trial cohort. This difference can be attributed to the lower efficacy of rPCSK9 compared with the activity of *PCSK9* in vivo. There are several explanations for this effect. First, in vivo, PCSK9 undergoes several posttranslational modifications such as Ser phosphorylation by *FAM20C*. This increases PCSK9 binding affinity to the LDLR. In vitro, there is an absence of phosphorylation by *FAM20C* in rPCSK9, which reduces the ability of PCSK9 to degrade *LDLR* (56). Second, high levels of cyclase-associated protein 1 (*CAP1*) are present in the liver as compared with in vitro conditions. *CAP1* has been shown to bind to PCSK9 to mediate endocytosis and lysosomal degradation of *LDLR*. Thus, lower levels of *CAP1* in vitro reduce the activity of *PCSK9* (57). For this reason, concentrations of PCSK9 supplemented in vitro cannot be compared with plasma PCSK9 levels.

Our molecular studies have been focused on DENV-targeted cells. The interpretation of cholesterol homeostasis at a systemic level in patients with dengue is probably more complex. Reduced circulating LDL-C levels have been associated with cases of severe dengue (58). Our PCSK9 finding also suggests that reduced plasma LDL-C levels in patients with severe dengue were not due to increased LDL-C uptake. Instead, it is possible that impaired cholesterol synthesis in the liver, which is known to be inflamed in patients with severe dengue (59), could have lowered cholesterol production. Alternatively, the associated increase in endothelial permeability could also have resulted in leakage of cholesterol molecules into the extravascular space and thus lowered plasma LDL-C levels (60, 61). Further studies will be needed to tease apart these possibilities.

Our study also has implications for dengue pathogenesis investigations. DENV infects myeloid cells in the lymph nodes, spleen, and liver where the microenvironments are physiologically hypoxic. Inflammation such as that triggered by infection, including DENV, can exacerbate hypoxia in these microenvironments as the generation of ROS depletes O_2_ levels further (62). That PCSK9 plays an important role in DENV infection under such low-oxygen conditions could thus not have been gleaned from conventional experimental studies on dengue that incubate virus and cells at ambient O_2_ levels. Likewise, the role of PCSK9 could also not have been deciphered in mouse studies. Mouse plasma cholesterol is transported in high-density lipoprotein (HDL) instead of LDL. The uptake of cholesterol in mice is therefore not dependent on *LDLR* (63, 64). PCSK9 would thus not have a significant impact on dengue pathogenesis in a mouse model.

In conclusion, our findings show how cholesterol metabolism in cells that reside in organs with low-oxygen concentrations is altered upon DENV infection to facilitate pathogenesis. Importantly, our study suggests that inhibition of PCSK9 activity using an inhibitory mAb or RNA interference approaches (25) could be safe therapeutic strategies for the treatment of patients with dengue.

## Methods

### Cells.

Huh7, BHK-21, and Vero cells were obtained from the American Type Culture Collection (ATCC) and cultured in DMEM (Gibco, Thermo Fisher Scientific) supplemented with 9% FCS (HyClone). Both cell lines tested negative for mycoplasma. When required, cells were adapted to 5% O_2_ in a fully humidified incubator supplied with 5% CO_2_ as well as nitrogen gas to reduce O_2_ levels for 24 hours before infection with DENV2.

### Monocytes and MoDCs.

PBMCs were isolated from a flavivirus-naive healthy donor. CD14^+^ monocytes were isolated from PBMCs using CD14 Microbeads (Miltenyi Biotec, 130-050-201) according to the manufacturer’s protocol. To obtain MoDCs, monocytes were cultured in 6-well tissue culture plates and supplemented with growth media (RPMI-1640, 10% FBS, 100 U/mL penicillin, 100 μg/mL streptomycin) containing 100 ng/mL IL-4 (eBioscience) and 50 ng/mL granulocyte macrophage–CSF (GM-CSF).

### Virus infection and plaque assays.

DENV2 (ST) is a clinical isolate obtained from Singapore General Hospital and passaged 6 times in Vero cells. DENV1-2402, DENV3-863, and DENV4-2270 are clinical isolates obtained from a previous clinical trial. For Huh7 infections, cells were infected with DENV for 2 hours at 37°C at a MOI of 1. Virus inoculum was then removed. After infection, cells are maintained in DMEM containing 9% FBS. Primary monocytes were infected with DENV-2 at a MOI of 10 for the duration of the experiment. MoDCs were infected with DENV-2 at a MOI of 1 for the duration of the experiment. Supernatants were collected at the specified time point and stored at –80°C before plaque assay quantification. A plaque assay was performed on BHK-21 cells using maintenance media with RPMI 1640 as previously described (65).

### qPCR.

RNA from cells was extracted with an RNeasy Kit (QIAGEN) followed by cDNA synthesis (QuantaBio) and real-time qPCR with SYBR (Roche) according to the manufacturer’s protocol. All RNA levels were measured relative to TATA box–binding protein (TBP). The primer sequences used were as follows: DENV, forward, TTGAGTAAACYRTGCTGCCTGTAGCTC and DENV, reverse, GAGACAGCAGGATCTCTGGTCTYTC; TBP, forward, TGTATCCACAGTGAATCTTGGTTG and TBP, reverse, GGTTCGTGGCTCTCTTATCCTC; LDLR, forward, GAATCTACTGGTCTGACCTGTCC and LDLR, reverse, GGTCCAGTAGATGTTGCTGTGG; PCSK9, forward, GACACCAGCATACAGAGTGACC and PCSK9, reverse, GTGCCATGACTGTCACACTTGC; SREBP2, forward, CTCCATTGACTCTGAGCCAGGA and SREBP2, reverse, GAATCCGTGAGCGGTCTACCAT; GLUT1, forward, TTGCAGGCTTCTCCAACTGGAC and GLUT1, reverse, CAGAACCAGGAGCACAGTGAAG; IFNβ, forward, CTTGGATTCCTACAAAGAAGCAGC and IFNβ, reverse, TCCTCCTTCTGGAACTGCTGCA; and CXCL10, forward, GGTGAGAAGAGATGTCTGAATCC and CXCL10, reverse, GTCCATCCTTGGAAGCACTGCA.

### LDLR flow cytometry.

After oxygen adaptation or DENV2 infection, cells were dissociated with Accutase (STEMCELL Technologies, 07920), washed with PBS, and fixed with 3% paraformaldehyde at 4°C for 30 minutes. Mouse anti-LDLR (1:300, R&D Systems) was then added for 30 minutes at 4°C. After further washing with PBS, anti–mouse Alexa Fluor 647 (Invitrogen, Thermo Fisher Scientific; 1:400) was added and incubate at 4°C for 30 minutes before analysis with the BD LSRFortessa Flow Cytometer.

### DIL-LDL.

DIL-labeled LDL (Thermo Fisher Scientific, L3482) was diluted in serum-free DMEM to a concentration of 1ug/mL. After oxygen adaptation or DENV2 infection, Huh7 cells were washed with PBS before addition of 1 μg/mL DIL-LDL. Cells were then incubated at 37°C for 3 hours, washed in PBS, and fixed in 3% paraformaldehyde (PFA) for 30 minutes before FACS analysis.

### Alirocumab.

Praluent (Sanofi US and Regeneron Pharmaceuticals), 150 mg/mL (alirocumab), was used for the experiments. The drug was stored at 4°C and diluted to a working concentration of 1 μM in DMEM.

### MTS assay.

CellTiter 96 AQueous One Solution Cell Proliferation Assay (MTS) was purchased from Promega (G3580). Briefly, MTS is a tetrazolium compound [3-(4,5-dimethylthiazol-2-yl)-5-(3-carboxymethoxyphenyl)-2-(4-sulfophenyl)-2H-tetrazolium)] used in a colorimetric assay to determine the number of viable cells. To assay the effects of statins, Huh7 cells were seeded overnight before treatment with statins for 24 hours at 5% O_2_. MTS was added for 2 hours and absorbance read at 490 nm.

### Western blotting.

Cells were washed once in PBS and resuspended in lysis buffer (1% Nonidet P-40, 150 mM NaCl, 50 mM Tris, pH8.0) in the presence of protease inhibitors (1:100, MilliporeSigma). Proteins in cell lysates were denatured at 95°C in loading buffer (Bio-Rad) and 2-mercaptoethanol (1:10) before separation by SDS-PAGE, transferred onto PVDF membranes (MilliporeSigma), and incubated with primary Abs followed by HRP-conjugated anti-rabbit (1:10,000, Abcam 6721) or anti-mouse (1:10,000, Dako P0447) antiserum. The following primary Abs were used: anti-LAMP1 (1:1000, eBioscience 6721), anti-STING (1:1000, CST 13647S), anti–p-STING (1:1000, CST 72971), anti-TBK (1:1000, CST 3504S), anti–p-TBK (1:1000, CST 5483S), and anti-calreticulin (1:1000, Abcam 2907). Blots were developed using ECL detection reagents (Amersham). The data shown are representative of 3 independent experiments. Quantification of protein densitometry was performed with ImageJ, version 1.47 (NIH).

### NanoString.

Extracted RNA (50 ng) was hybridized to the NanoString nCounter (NanoString Technologies) human inflammation panel at 65°C for 24 hours. Hybridized samples were quantified using the nCounter Sprint profiler (NanoString Technologies). Data were analyzed using the nSolver Analysis Software (NanoString Technologies). Subsequent pathway analysis was performed using GO Biological Pathways analysis.

### ER isolation and cholesterol quantification.

Isolation of ER from Huh7 cells was performed as previously described (66). Cholesterol quantification was performed using the Amplex Red Cholesterol Assay Kit (A12216, Thermo Fisher Scientific) according to the manufacturer’s protocol. Cholesterol levels were normalized to the densitometry of calreticulin to account for any difference in ER concentrations.

### PCSK9 quantification.

Huh7 cells were centrifuged at maximum speed at 4°C for 10 minutes to remove any cellular debris. Supernatant was stored at –80°C before quantification. PCSK9 quantification was performed at 1:10 dilution with the Human PCSK9 ELISA Kit (Abcam, 209884) according to the manufacturer’s protocol.

### Patient plasma PCSK9 quantification.

Plasma concentrations of PCSK9 were measured at 3 time points: study enrollment, 48 hours later, and follow-up 10–14 days after illness onset. These were performed at the OUCRU laboratories in Ho Chi Minh City, using a Human Magnetic Luminex Assay (Premixed Multiplex Kit, LXSAHM-05) on a Luminex 200 analyzer, according to the manufacturer’s specifications (R&D Systems).

### Clinical study.

A nested case-control study was performed using stored plasma samples from a prospective study that enrolled 316 patients from both outpatient facilities and inpatient wards at the Hospital for Tropical Disease (HTD) (Ho Chi Minh City, Vietnam) and the National Hospital for Tropical Diseases (NHTD) (Hanoi, Vietnam) (40). From this cohort, we randomly selected 111 patients including 56 with plasma leakage (29 patients with grade 2 and 27 patients with grade 1) compared with 53 patients without evidence of leakage. Patients were included if they had sufficient plasma stored for more than 1 time point and all the clinical data required for the grading of plasma leakage. Patients were classified into 3 predefined categories of increasing disease severity as indicated by plasma leakage: grade 0, no evidence of leakage; grade 1, a 15%–20% increase in HCT and/or fluid accumulation; grade 2, evidence of severe leakage including an HCT increase of greater than 20%, DSS, or compromised respiratory function. Dengue diagnostics for the NS1 test (Platelia ELISA, Bio-Rad), Ig serology, and RT-PCR were described in the original publication (40).

### Statistics.

In vitro experiments were replicated 3 times, each with a minimum of 3 biological replicates. Representative data from 1 of these 3 independent experiments are shown in the figures. A 2-tailed, unpaired *t* test was performed to compare between the means of 2 conditions using GraphPad Prism, version 8.0 (GraphPad Software). Statistical analysis for data sets with more than 2 groups was performed with 1-way ANOVA corrected with Tukey’s multiple comparisons test. For all data sets, a *P* value of less than 0.05 was considered significant. Statistical analysis for patient clinical parameters was performed with 1-way ANOVA corrected for multiple comparisons with Dunnett’s test against group 0 (control).

### Study approval.

Ethics approval for the clinical study “An investigation into the pathophysiology of disease progression in dengue in Vietnam” was obtained from the Oxford Tropical Research Ethics Committee (OXTREC reference: 1030-13) and the ethics review committee at the NHTD (Ho Chi Minh City, Vietnam). PBMCs were isolated from a healthy donor under a protocol approved by the National University of Singapore Institutional Review Board (IRB B-15-227).

## Author contributions

ESG, SY, and EEO designed the study. ESG and HCT performed the in vitro experiments. DHTL performed the PCSK9 Luminex assay on samples from the Vietnamese cohort. BW, SY, and TTH led the clinical studies in Vietnam. ESG and EEO analyzed the data and wrote the first version of the manuscript. ESG, NGS, BW, SY, and EEO reviewed and revised the manuscript.

## Supplementary Material

Supplemental data

## Figures and Tables

**Table 2:**
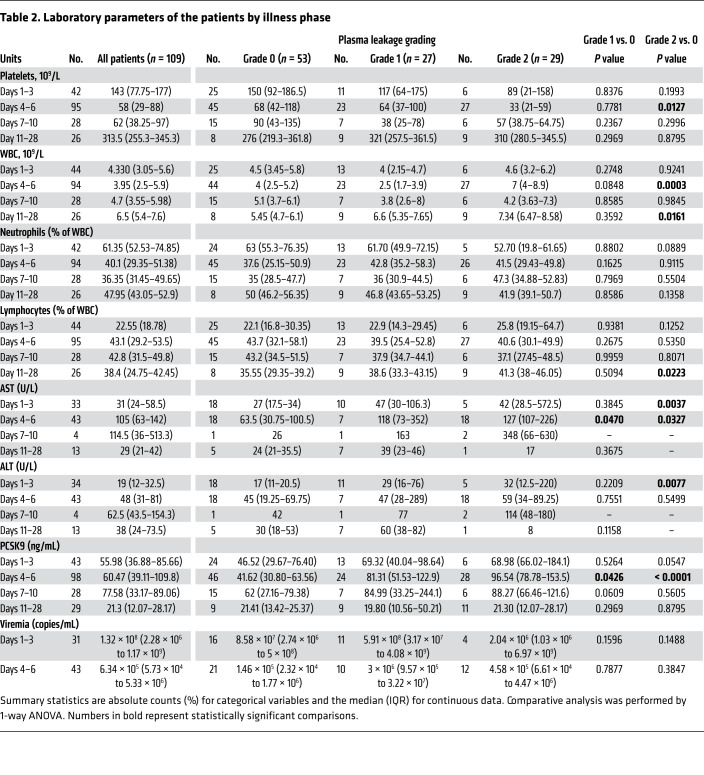
Laboratory parameters of the patients by illness phase

**Table 1:**
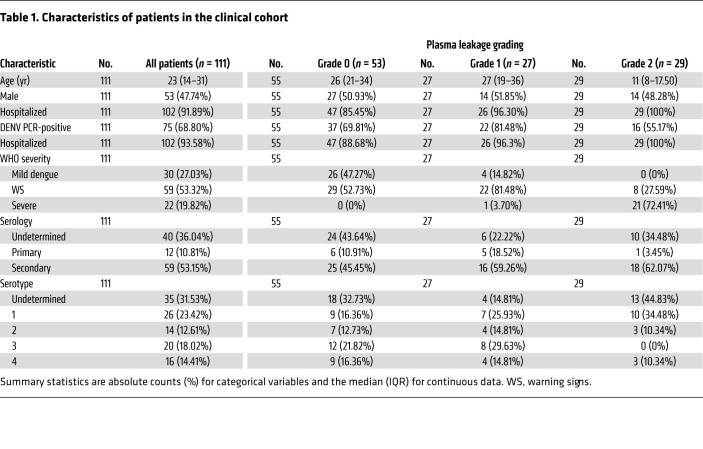
Characteristics of patients in the clinical cohort

## Version 1 07/09/2020

In-Press Preview

## Version 2 08/31/2020

Electronic publication

## Version 3 10/01/2020

Print issue publication
