# Supplementary material for: Dengue virus induces PCSK9 expression to alter antiviral responses and disease outcomes
Source: J Clin Invest. 2020 Aug 31;130(10):5223–34. doi: 10.1172/JCI137536 (PMC7524462; doi:10.1172/JCI137536)
Supplement: Supplemental data [file jci-130-137536-s112.pdf]

Supplementary Figures

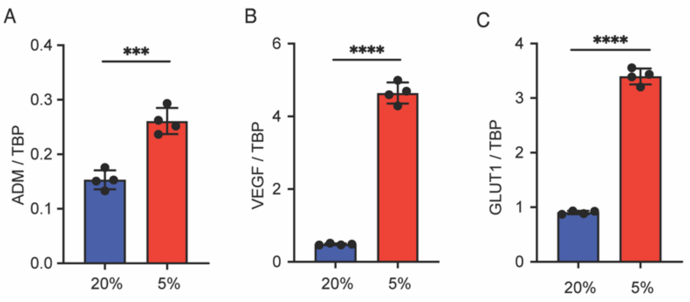

Supplementary Figure 1: Hypoxia upregulates cholesterol biosynthesis genes **(A)** *ADM* mRNA expression in normoxic (blue) or hypoxic (red) Huh7 cells 24 hours post oxygen adaptation. **(B)** *VEGF* RNA expression in normoxic (blue) or hypoxic (red) Huh7 cells 24 hours post oxygen adaptation. **(C)** *GLUT1* RNA expression in normoxic (blue) or hypoxic (red) Huh7 cells 24 hours post oxygen adaptation. Experiments were replicated 3 times, each with a minimum of 3 biological replicates. Representative data from 1 of these 3 independent experiments are shown in the figures. Data in (A-C) represents mean ± SD. ***P<0.001, ****P<0.0001 (unpaired t-test)

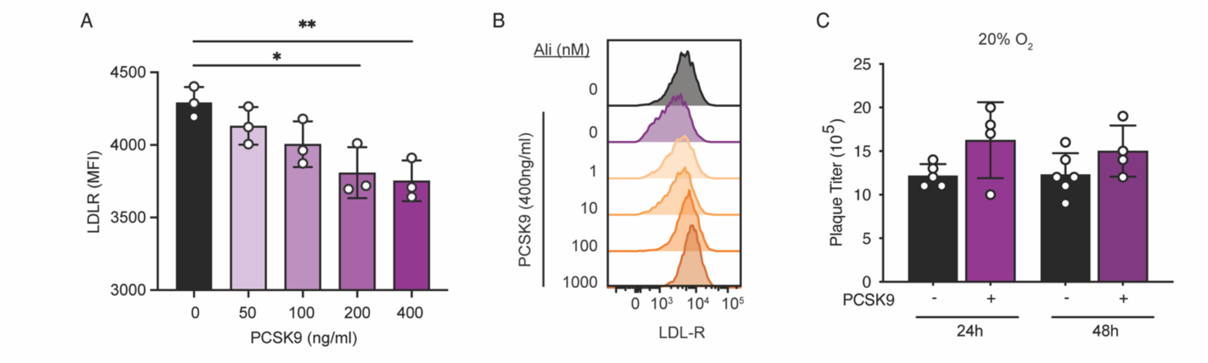

Supplementary Figure 2: PCSK9 alters LDLR expression

(A) Mean fluorescence intensity (MFI) of LDLR in hypoxic Huh7 cells supplemented with increasing concentrations of recombinant PCSK9. **(B)** Mean fluorescence intensity (MFI) of LDLR in hypoxic Huh7 cells supplemented with 400ng/ml PCSK9 and varying concentrations of alirocumab. **(C)** Plaque titers in normoxic Huh7 cells at 24 and 48hpi with or without PCSK9 supplementation. Mean fluorescence intensity (MFI) of LDLR in hypoxic Huh7 cells supplemented with 400ng/ml PCSK9 and varying concentrations of alirocumab. Experiments were replicated 3 times, each with a minimum of 3 biological replicates. Representative data from 1 of these 3 independent experiments are shown in the figures. Data in (A and C) represents mean ± SD. *P<0.05, **P<0.01 (unpaired t-test)

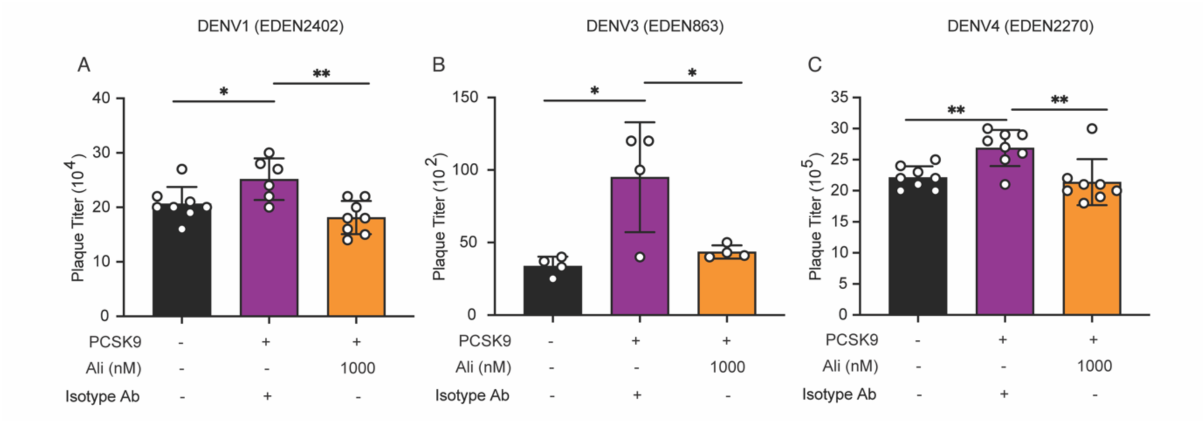

Supplementary Figure 3: PCSK9 alters DENV infection of different serotypes (A-C) Plaque titers in hypoxic Huh7 cells 48 hours post-infection with DENV1 (A), DENV3 (B) and DENV4 (C). Cells were cultured without (black), with supplementation of 400ng/ml *PCSK9* (purple) or PCKS9 with alirocumab (orange) for 24 hours prior to DENV infection. Experiments were replicated 3 times, each with a minimum of 3 biological replicates. Representative data from 1 of these 3 independent experiments are shown in the figures. Data in (A-C) represents mean ± SD. *P<0.05, **P<0.01 (unpaired t-test)

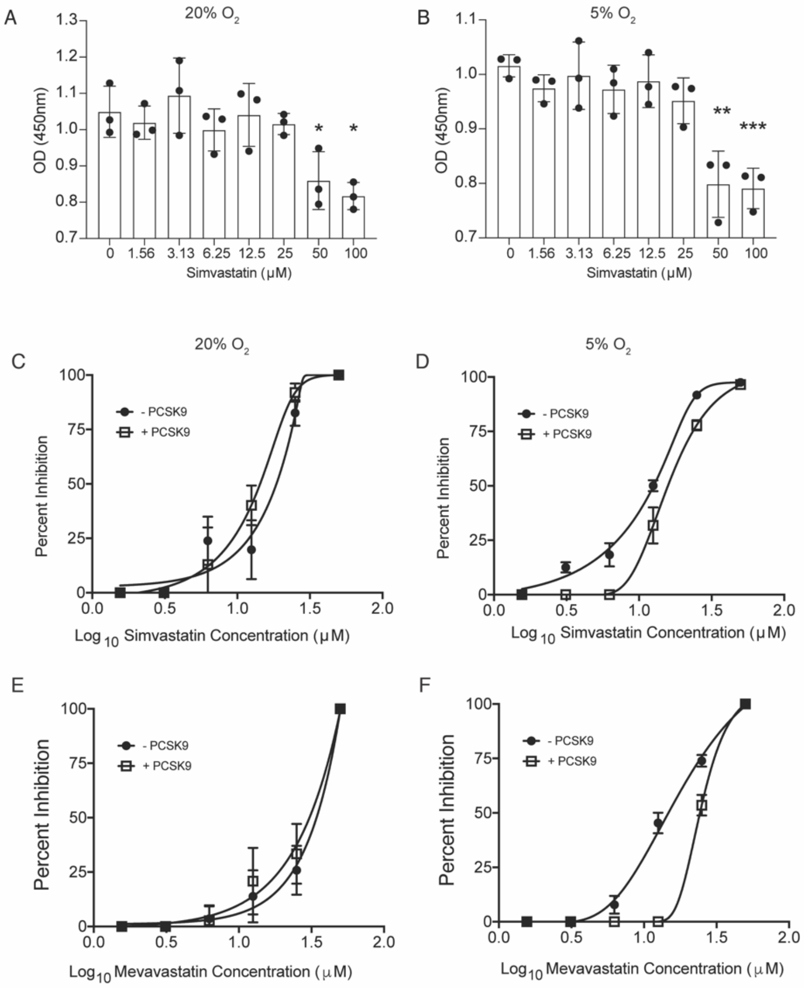

Supplementary Figure 4: PCSK9 alters antiviral activity of statins under hypoxic conditions. (A-B) MTS OD values of normoxic (A) and hypoxic (B) Huh7 48 hours post simvastatin treatment. **(C-D**) Dose response curves for EC50 analysis of normoxic (C) and hypoxic (D) Huh7 cells 48hpi with DENV2 treated with varying doses of simvastatin, with or without PCSK9 supplementation. **(E-F**) Dose response curves for EC50 analysis of normoxic (E) and hypoxic (F) Huh7 cells 48hpi with DENV2 treated with varying doses of mevastatin, with or without PCSK9 supplementation. Experiments were replicated 3 times, each with a minimum of 3 biological replicates. Representative data from 1 of these 3 independent experiments are shown in the figures. Data in (A-B) represents mean ± SD. *P<0.05, ***P<0.001, ***P<0.001 (unpaired t-test)

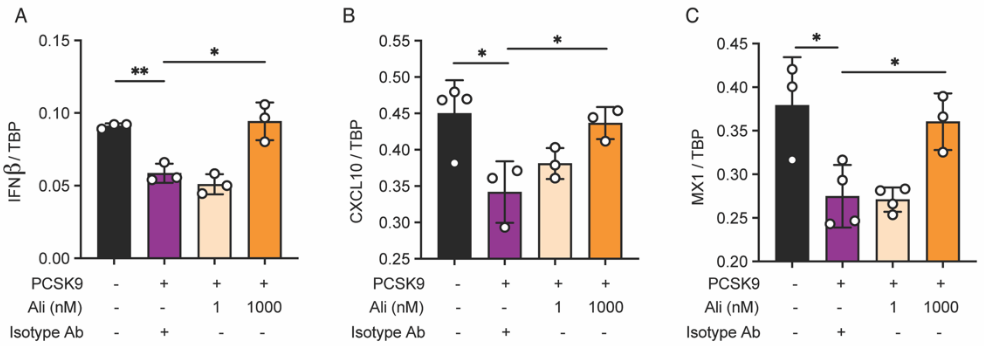

Supplementary Figure 5: PCSK9 suppresses induction of type-I IFN during DENV infection. **(A-C)** mRNA expression of *IFNb* (A), *CXCL10* (B) and *MX1* (C) in hypoxic Huh7 cells without (black) or with PCSK9 supplementation (purple) 6 hours post DENV2 infection. Cells were with increasing doses of alirocumab (orange) for 24 hours prior to DENV2 infection. Experiments were replicated 3 times, each with a minimum of 3 biological replicates. Representative data from 1 of these 3 independent experiments are shown in the figures. Data in (A-C) represents mean ± SD. *P<0.05, **P<0.01 (unpaired t-test)

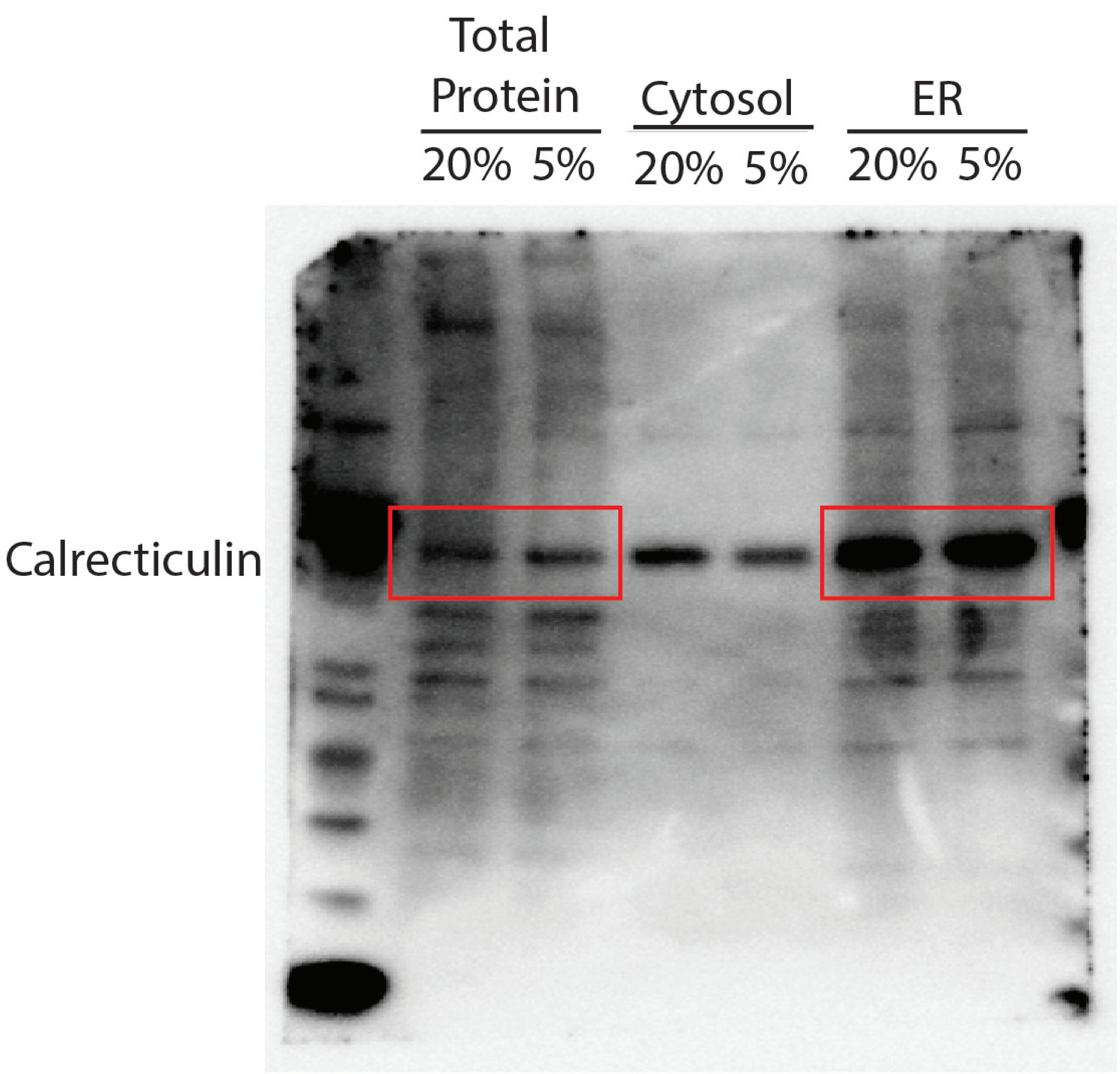

Full unedited gel for Figure 4A and 4B.

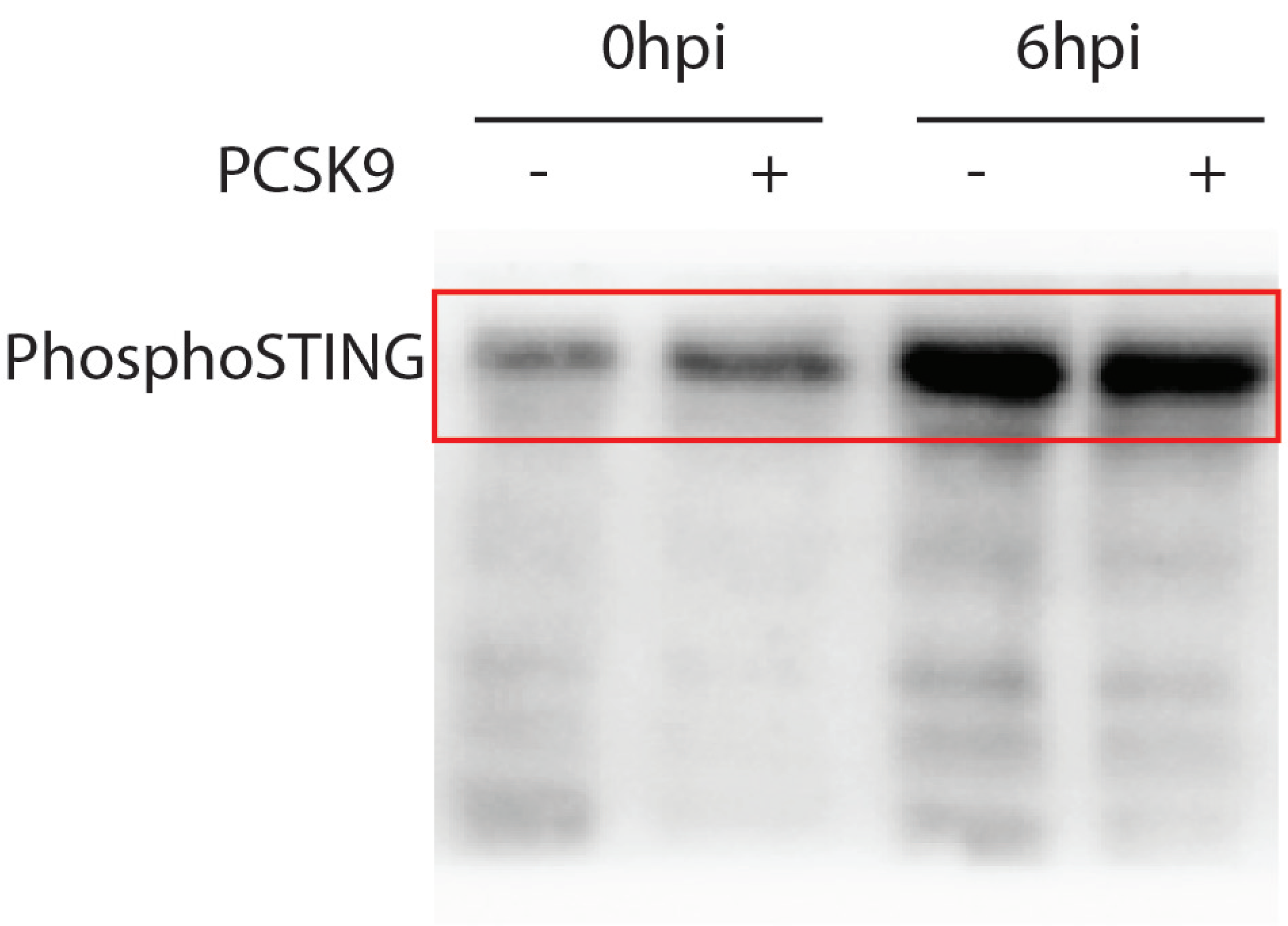

Full unedited gel for Figure 4C

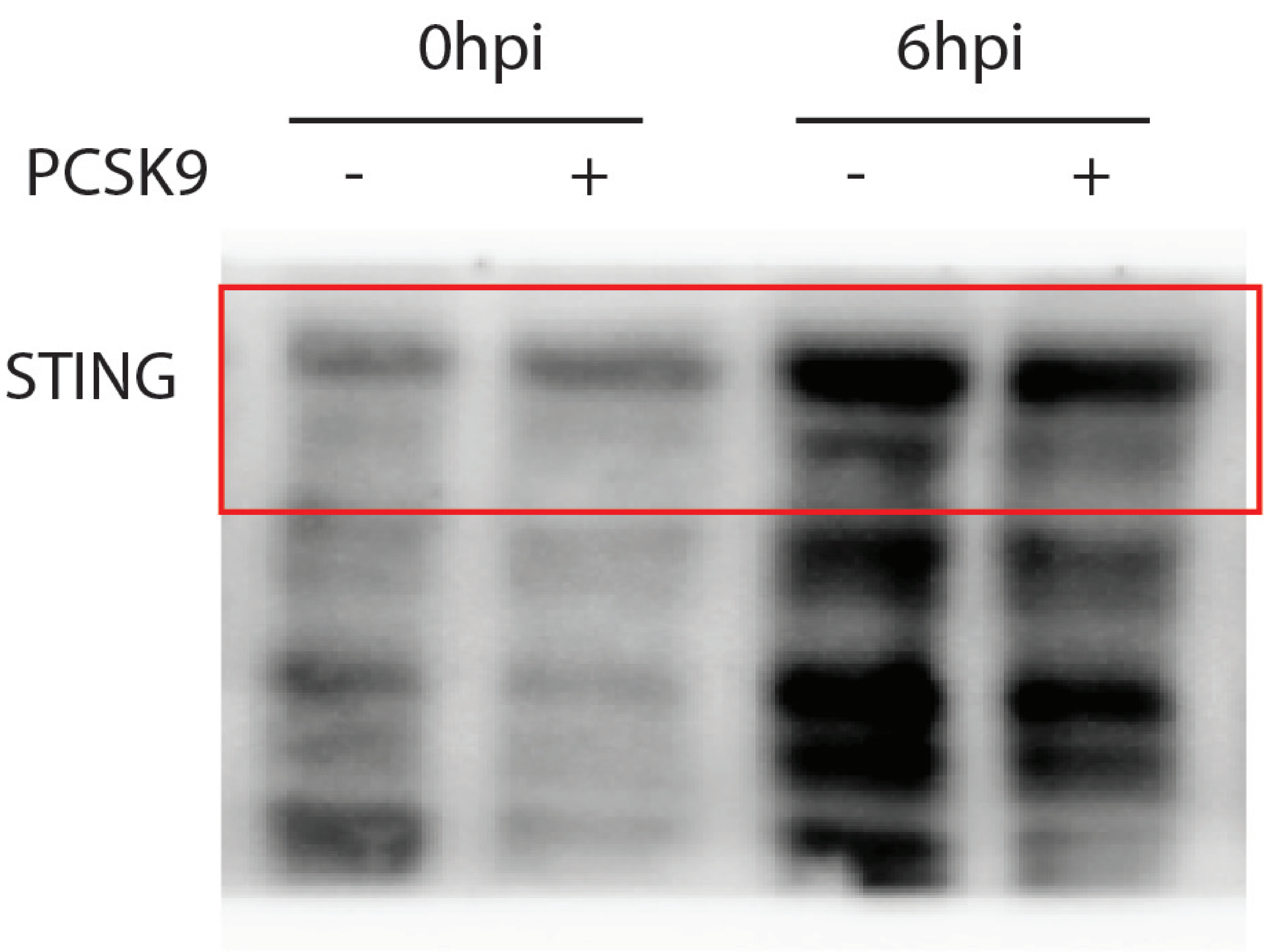

Full unedited gel for Figure 4C

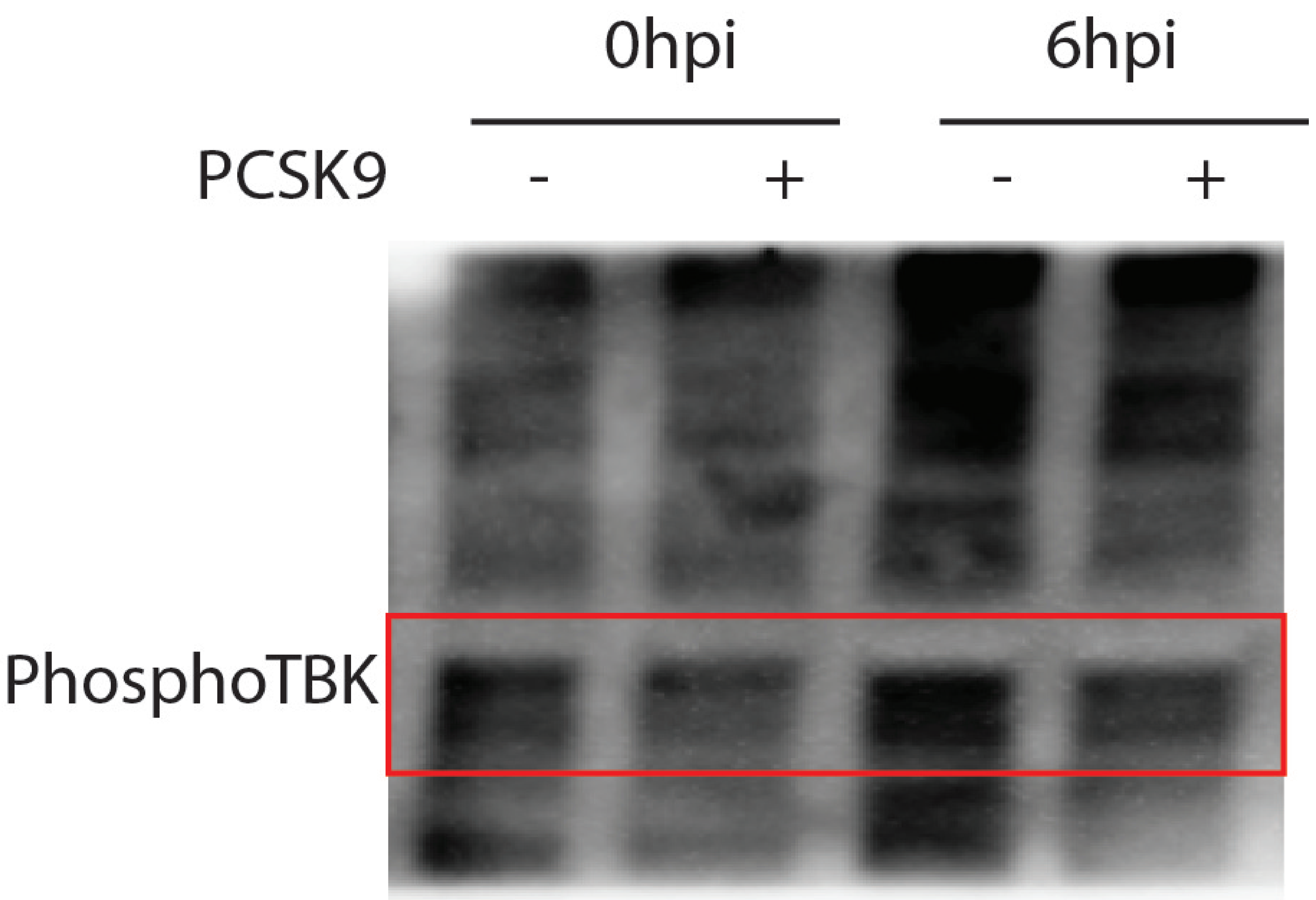

Full unedited gel for Figure 4C

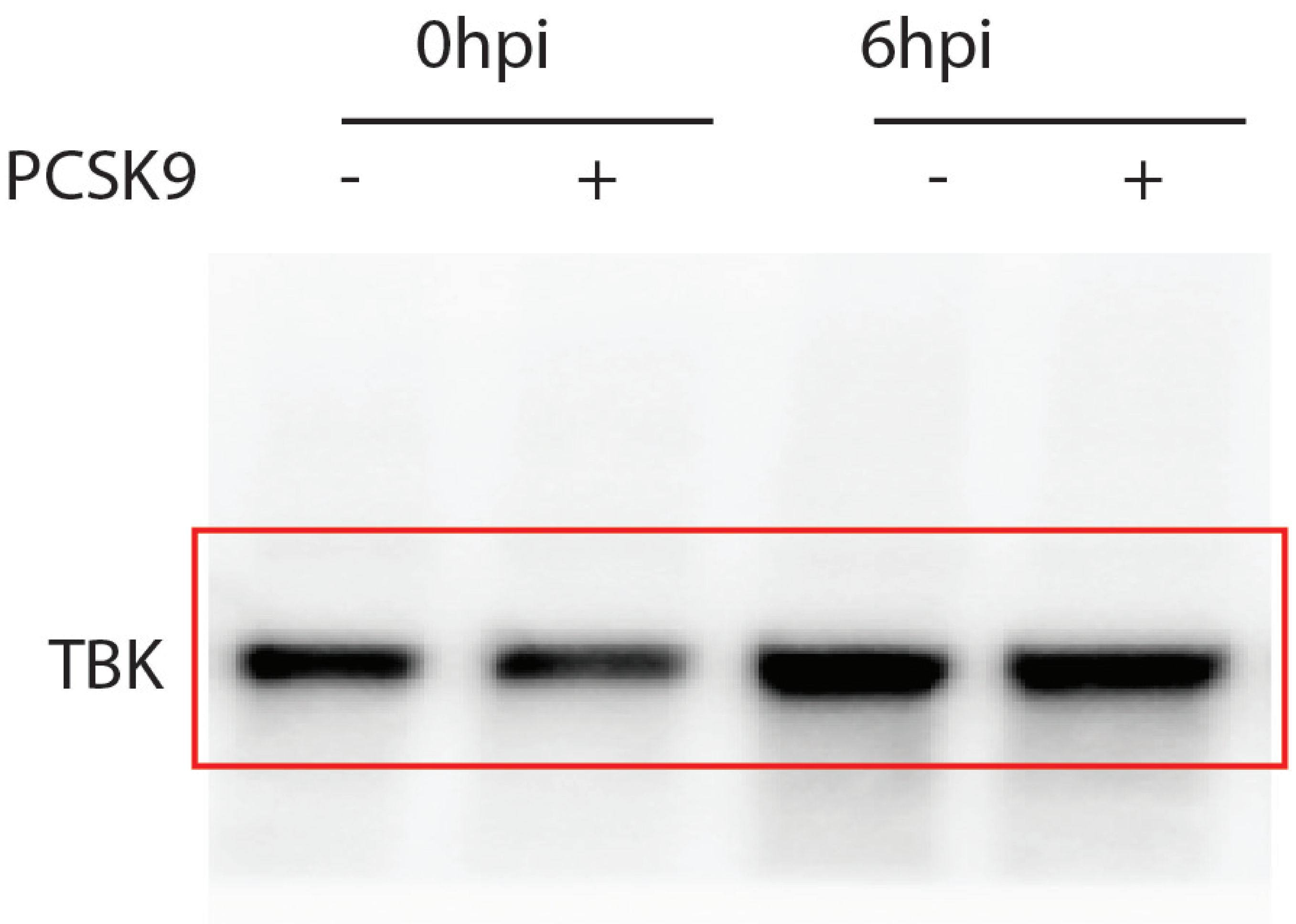

Full unedited gel for Figure 4C

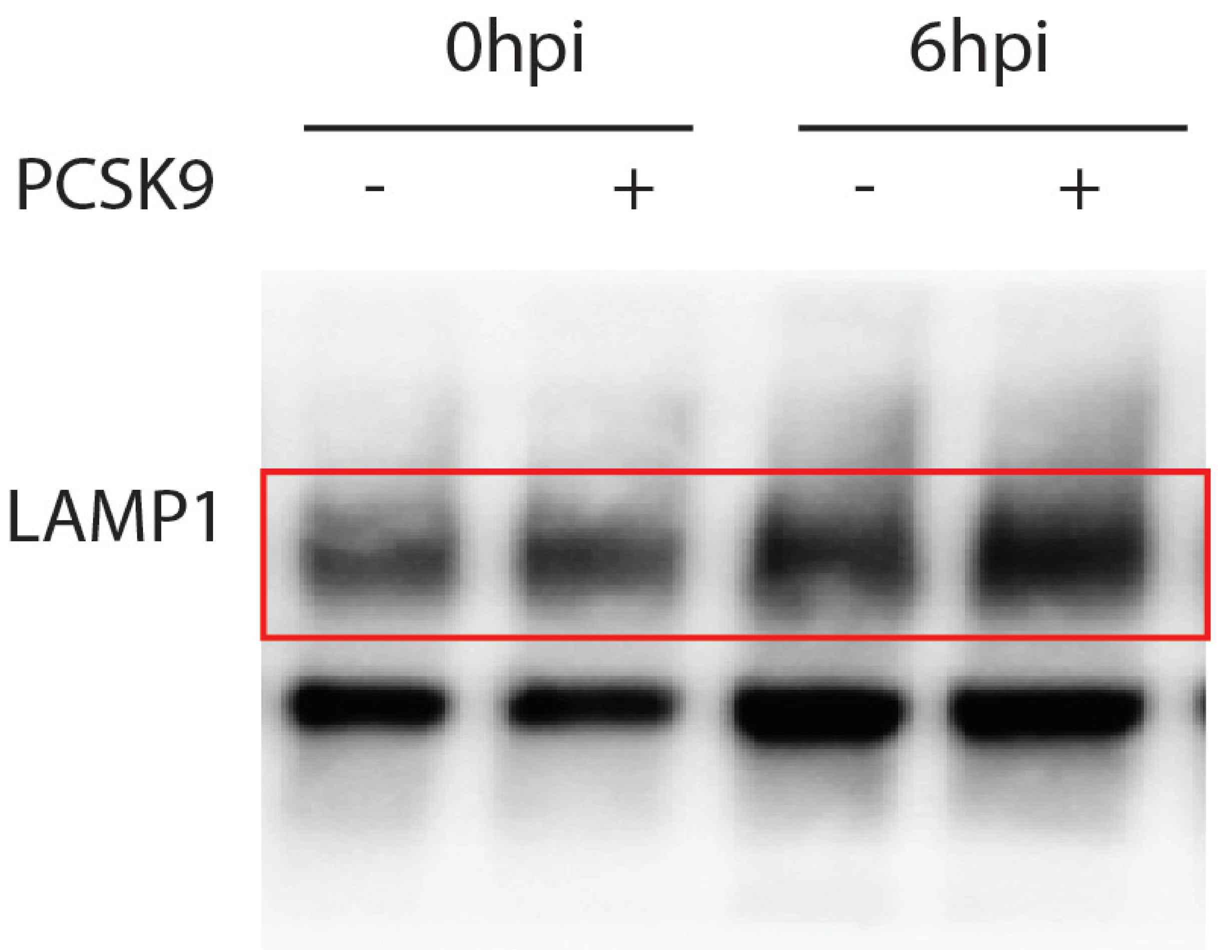

Full unedited gel for Figure 4C
